# The Effect of a Small Amount SiO_2_ on Sintering Kinetics of Tetragonal Zirconia Nanopowders

**DOI:** 10.1186/s11671-017-2178-6

**Published:** 2017-06-08

**Authors:** Marharyta Lakusta, Igor Danilenko, Tetyana Konstantinova, Galina Volkova, Igor Nosolev, Oksana Gorban, Susanna Syniakina, Valery Burkhovetskiy

**Affiliations:** Material Science Department, Donetsk Institute for Physics and Engineering (DIPE) named after O.O. Galkin of the NAS of Ukraine, Nauky av., 46, Kiev 03028 Ukraine

**Keywords:** Zirconia nanopowders, Sintering kinetics, Silica additive, Sintering mechanisms, Initial sintering stage

## Abstract

In the present paper the sintering behavior of 3 mol% yttria-stabilized zirconia (3Y-TZP) with and without small amount (0.2 wt %) of SiO_2_ additive was investigated. It has been studied the silica impact which was added in two ways (co-precipitation and mechanical mixing) on sintering kinetics of 3Y-TZP nanopowders at the initial sintering stage. It was found the silica additive leads to the changing in the predominant sintering mechanism at the initial sintering stage from volume (VD) to the grain boundary diffusion (GBD) in nanopowders obtained by co-precipitation. It was shown that the way of silica addition also significantly influence the sintering kinetics of 3Y-TZP. In case of nanopowders with silica additive obtained by mixing method, sintering process occurred due to the predominance of VD mechanism. It was found that the silica additive and the mechanical activation leads to the acceleration of the sintering process.

## Background

It is a well-known fact that zirconia is really remarkable for a wide range and combination of physical and mechanical properties, such as high fracture toughness; high strength and hardness; biocompatibility; ionic conductivity; radiation and chemical resistance [1]. A lot of properties combined in one material, zirconia. It is possible due to the ability of zirconia to phase transformations [2]. Zirconia can exist in three states: a monoclinic state, a tetragonal state, and a cubic state. These states can be stabilized by adding such additives as Y_2_O_3_, MgO, CaO [3]. Yttria-stabilized tetragonal zirconia (Y-TZP) has been known as an important structural ceramic and is used for products of grinding media, the optical fiber connector and precision parts. In all cases of using zirconia nanopowders, the producer of ceramics parts need to know the optimal pressure-temperature-time regimes for obtaining dense or porous nanostructure ceramics. The advantage of nanopowders is the possibility of low temperature sintering and as a result the ceramic structure homogeneity. It is known that granulometric (size and shape of particles and the size of aggregates and agglomerates), phase and chemical composition of starting powders as well as the same characteristic of agglomeration as agglomerate “hardness” determine the compactions and sintering regime. The agglomeration is conditioned by van der Waals forces between particles. If these forces are weak the agglomerates are referred to as “soft” agglomerates. These agglomerates can be easily broken in a liquid medium by ultrasonic, or/and dispersants additions. In contrast, strong forces between particles due to high temperature calcinations or incorrect chemical additions result in “hard” agglomerates. In this case, it is too high to realize the benefits of the nanosized primary crystallites. The high sintering temperature leads to bimodal grain size distribution and phase separation in zirconia ceramics. The prevention of hard agglomeration is a one of the basic aims in nanopowders synthesis process as well as uniform particles shape and narrow size distribution [2].

As for the additives that influence the zirconia nanopowders structure (Al_2_O_3_, NiO, Cr_2_O_3,_ SiO_2,_ GeO_2_) it has become possible to obtain new ceramics with specific properties. The impact of various additives on the sintering kinetics has been investigated by many researchers [3–7]. One of the well-known researchers in the field of research of the effect of different additives on the tetragonal zirconia, Matsui has reported that the silica additive accelerates the sintering process because the sintering mechanism is changed from grain boundary to volume diffusion by silica addition [3, 4].

In the present paper the impact of small amount of slightly soluble SiO_2_ additive on the kinetics of the initial sintering stage of ceramics based on 3Y-TZP has been studied. In our previously investigation of the sintering kinetics of 3Y-TZP nanopowders we have got contradictory results using nanopowders which were obtained in DIPE laboratory with the same chemical composition. Our results did not agree with the results of studies conducted over the TZ-3Y nanopowders of Tosoh company production. The reason for the difference of predominant sintering mechanisms at the initial stage has been identified in our study [8]. It was concluded that this result is due to the influence of mechanical activation on powders structure, phase composition and the sintering kinetics of 3Y-TZP.

## Methods

For the investigation it has been chosen 3Y-TZP nanopowder (3 mol% Y_2_O_3_-stabilized tetragonal zirconia) obtained in the DIPE of the NASU (Ukraine) by co-precipitation method. It was used a chloride technology and the addition of 0.2 wt% SiO_2_ to produce these nanopowders. The preparation technique has been described in detail in paper [9]. The silica additive was added in two ways:

1. by co-precipitation method was obtained the nanopowders: with and without the addition of the silica 3Y-TZP- 0,2 wt % SiO_2_; 3Y-TZP, respectively;
2. by the mixing method was obtained nanopowders with silica and with mechanical activation for 4 and 8-h milling PMM4-3Y-TZP-0,2 wt % SiO_2_ and PMM8-3Y-TZP-0,2 wt % SiO_2_ (PMM4 and PMM8 abbreviations were marked for powders name mixing and milling for 4 and 8 h).

To separate the impact of the silica and the effect of mechanical activation were obtained 3Y-TZP with the same milling time 4 and 8 h PM4-3Y-TZP and PM8-3Y-TZP (the PM4 and PM8 abbreviation means milling for 4 and 8 h).

All the obtained nanopowders were calcinated at 1000 °C within 2 h. Then in the last two cases nanopowders were mechanically milled in a planetary mill. Thereafter, all nanopowders were pressed at 300 MPa and sintered to the temperature of 1500 ^0^ C with different heating rates of 2.5, 5, 10, and 20 °C/min in the dilatometer (NETZSCH DIL 402 PC). The shrinkage data of the sintering powder compacts was obtained using a dilatometer which was calibrated using a standard Al_2_O_3_. Thermal expansion of each sample was corrected with the cooling curve by the method described in [7, 9]. The shrinkage occurred isotropically. The final density of sintered samples was measured using the Archimedes method.

The characteristics of all nanopowders were evaluated by X-ray diffraction (XRD) employing a Dron-3 diffractometer with Cu-K α radiation. Fitting and analysis of the XRD curves were made by Powder Cell software for Windows version 2.4. Crystallite sizes (d_XRD_) were calculated from the line broadening of the X-ray diffraction peaks using the Debay–Scherrer Equation [10]. The specific surface area and crystallite sizes (d_BET_) were measured by the Brunauer-Emmett-Teller (BET) method on the “SORBI-4” device. The nanopowders structures were also studied by transmission electron microscope TEM (Jem 200A, JEOL, Japan) and observed average particle size was compared with the value obtained by XRD. The nanopowders structure formation was investigated by methods FTIR (model TENSOR 27, BRUKER) and TG-DTA (model Linses 1600, Germany). The histograms of particle size were obtained from the measurements of 200-250 particles in TEM images. Agglomeration degrees (agglomeration factor) of all nanopowders were calculated as described in papers [2, 11, 12].

The chemical composition and EDX mapping analysis of synthesized materials were checked by energy dispersive spectroscopy (EDS) analysis (JSM6490 LV JEOL, Japan with EDX analyses, Oxford, England). The microstructures of the ceramics were studied by scanning electron microscopy (JSM 6490LV JEOL) after polishing of the surfaces as well as fractured surfaces.

To analyze the dilatometric data of initial sintering stage it was used the standard constant rate of heating (CRH) technique [13–15]. This analytical method is applicable only for analyzing the initial sintering stage (it is not more than 4% of relative shrinkage). At this temperatures range interparticle contacts starts forming and growing, but grain growth insignificant yet. To define the activation energy of sintering was used an analytical Eq. (1) derived by Wang and Raj:

1 $$ \mathrm{T}\cdot \mathrm{c}\frac{\mathrm{d}\uprho}{\mathrm{d}\mathrm{T}}=\frac{1}{{\mathrm{F}}^{\prime}\left(\uprho \right)}\cdot \frac{\mathrm{K}\upgamma \Omega \mathrm{D}}{{\mathrm{kTa}}^{\mathrm{p}}}\cdot \exp \left(-\frac{\mathrm{Q}}{\mathrm{RT}}\right) $$

Here, *T* is the temperature; *c* – the heating rate; *ρ* – the density; *F*’(ρ) – the density function than depends on *n*; K-the numerical constant – *γ* the surface energy; Ω – the atomic volume; D – the diffusion coefficient, k – the Boltzmann constant, a – the particle radius; parameters *n* and *p* are the order depending on diffusion mechanism, Q – the activation energy and R – the gas constant. Using the slope S_1_ of the Arrhenius-type plot of ln[T(dT/dt)(dρ/dT)] against 1/T at the same density, the *Q* is expressed as

2 $$ \mathrm{Q} = \hbox{-} {\mathrm{RS}}_1 $$

To define the parameter, *n* was used Yang and Cutler’s Eq. (2). This helped determine the sintering mechanism at the initial sintering stage.

3 $$ \frac{\mathrm{d}\left(\Delta \mathrm{L}/{\mathrm{L}}_0\right)}{\mathrm{d}\mathrm{T}}=\left(\frac{{\mathrm{K}\upgamma \Omega \mathrm{D}}_0\mathrm{R}}{{\mathrm{ka}}^{\mathrm{p}}\mathrm{cQ}}\right)\cdot \left(\frac{\mathrm{nQ}}{{\mathrm{RT}}^{2-\mathrm{n}}}\right)\cdot \exp \left(-\frac{\mathrm{nQ}}{\mathrm{RT}}\right) $$

Here, ΔL = (L_0_-L) is the change in length of the specimen; *c* = dT/dt is the heating rate and *D*_0_ is the pre-exponential term defined as *D* = D_0_exp(-Q/RT). Using the slope S_2_ of Arrhenius-type plot of ln[T^2-n^ d(ΔL/L_0_)/dT] against 1/T was found:

4 $$ \mathrm{n}\mathrm{Q}=\hbox{-} {\mathrm{RS}}_2 $$

Considering that if *n* = 1, this means that the viscous flow mechanism dominates. If *n* = 1/2, the volume diffusion mechanism dominates and if *n* = 1/3, the grain boundary diffusion mechanism dominates.

## Results and Discussion

**Fig. 1:**
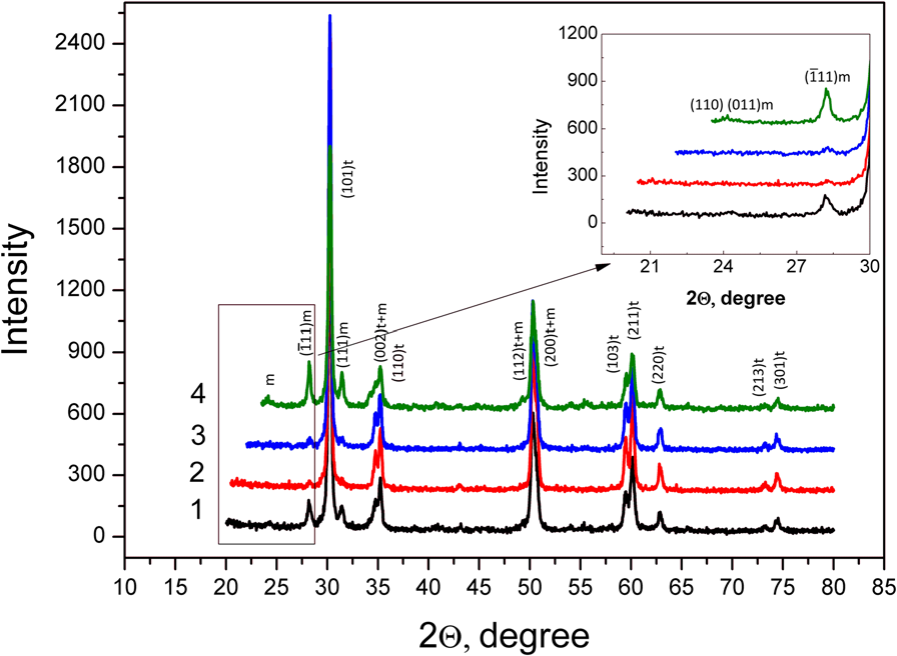
The XRD patterns of nanopowders with and without silica additive: 1- PMM8-3Y-TZP-0.2 wt% SiO_2_; 2 -3Y-TZP-0.2 wt% SiO_2_; 3-3Y-TZP; 4 - PM8-3Y-TZP

The nanopowders characteristics are shown in Table 1. The XRD spectra of nanopowders synthesized by mixing and co-precipitation technique presented on the Fig. 1. It is shows that the silica additive and a mechanical milling hardly affected the crystallites size and phase composition of the nanopowders. And a mechanical activation during 8 h leaded to a slight increase in the monoclinic phase amount. Figure1 confirms the fact that silica additive was not found as a separate phase so it can be supposed that in case of co-precipitation the additive in such a small amount enters the solid solution. The lattice parameters are decrease insignificantly in nanopowder 3Y-TZP-0.2 wt% SiO_2_ obtained by co-precipitation because the silica radius (*r* (Si^4+^) = 0.040 nm) is less than zirconia radius (r (Zr^4+^) = 0.0720 nm). In case of mechanically milled nanopowders with a small amount of silica and without silica, the decreasing in the lattice parameters is probably caused by milling (Table 2).

**Table 1:** The X-ray analysis results

No.	Nanopowders composition	Region of coherent scattering, nm	The phase composition, % M-phase	Lattice parameters, nm
1	3Y-TZP	31.5	3%M + 97%T	*a* = 5.09801 *c* = 5.17396
2	PM4- 3Y-TZP	31.5	8%M + 92%T	*a* = 5.09565 *c* = 5.16945
3	PM8- 3Y-TZP	28	15,5%M + 84,5%T	*a* = 5.09516 *c* = 5.16670
4	3Y-TZP-0.2 wt % SiO2	31.5	1,3%M + 98,7%T	*a* = 5.09555 *c* = 5.16953
5	PMM4-3Y-TZP-0.2 wt % SiO2	28.5	6% M + 94%T	*a* = 5.09561 *c* = 5.16940
6	PMM8-3Y-TZP-0.2 wt % SiO2	26.5	10% M + 90%T	*a* = 5.09510 *c* = 5.16668

*T* tetragonal phase, *M* monoclinic phase

**Table 2:** The effect of silica additive and mechanical activation on the particle sizes and agglomeration degree (agglomeration factor F_a_, %) of investigated nanopowders

No.	Nanopowders	d_XRD,_ nm	d_BET,_ nm	d_m,_ nm	S_BET_, g/m^2^	F_a_, %
1	3Y-TZP	31.5	70	33.16	14.2	57
2	4PM- 3Y-TZP	31.5	68	35.92	14.5	52
3	8PM- 3Y-TZP	28	50	25.92	20.0	51
4	3Y-TZP-0.2 wt % SiO_2_	31.5	85	36.92	12.0	57
5	4PMM-3Y-TZP-0.2 wt % SiO_2_	28.5	51	46.5	19.6	10
6	8PMM-3Y-TZP-0.2 wt % SiO_2_	26.5	48	46.42	20	4

**Fig. 2:**
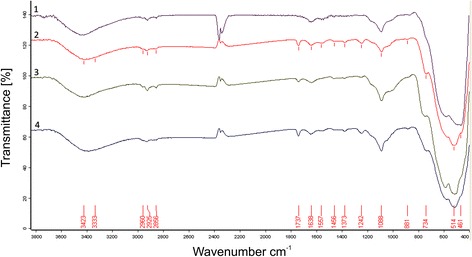
The FTIR spectra of nanopowders: 1- 3Y-TZP; 2 - PM8-3Y-TZP; 3- PMM8-3Y-TZP-0.2 wt% SiO_2_; 4- 3Y-TZP-0.2 wt% SiO_2_

FTIR spectra of the studied systems are shown in Fig.2. In the range 3700–3200 cm^-1^ and 1700–1300 cm^-1^ absorption bands appeared that correspond to valence and deformation vibrations of OH bonds of water molecules and hydroxyls which coordinated on the nanoparticles surface, respectively. The absorption bands appearing in the range 1200–1000 cm^-1^ are related to the surface vibrations of Zr = O (OH) and SiOH groups. The absorption bands observed below 1000 cm^-1^correspond to vibrations Zr-O-Zr and O-Zr-O bonds zirconia lattice.

In the range of stretching vibrations of Zr-O lattice bonds a number of bands with frequencies at 734 cm^-1^, 590 cm^-1^, 514 cm^-1^, and 461 cm^-1^ appear in the IR spectrum of the investigated powders. For the ZrO_2_-3 mol. % Y_2_O_3_ initial system three peaks with frequencies of 590 cm^-1^, 514 cm^-1^, and 461 cm^-1^ appear in the IR spectrum of this range, which indicates the formation of a predominantly tetragonal phase in this system [16]. For mechanical (milling) and/or chemically (the introduction of SiO_2_) of the modified oxide system, an absorption band with a frequency of 734 cm^-1^ appears in the IR spectrum, which corresponds to the Zr-O bonding of the monoclinic phase oriented in the ZrO_δ_polyhedra, where δ equals 4 or 6 [16]. An analysis of the qualitative picture of the IR spectrum in this range demonstrates changing in the ratio of the peaks intensity that correspond to Zr-O bonds oriented in different planes of different phases. Thus, in the IR spectrum of physically and chemically modified systems, the contribution of the high-frequency band in the high-frequency range of 514 cm^-1^ is enhanced in contrast to the initial system. For this system the most intense band is the absorption band at 461 cm^-1^ (as shown in Fig. 1). This indicates the appearance in the modified system of faces with a lower coordination number of surface atoms of zirconium and oxygen in relation to volumetric [17]. onds a number ofReduction of the coordination number of zirconium and oxygen atoms can be the result of the appearance of surface defects induced by physical and/or chemical action on the initial system. The decrease in the coordination number of zirconium and oxygen on the surface faces and the appearance of the monoclinic phase lead to an increase in the surface energy of the nanoparticles ${E}_{0, surf}^t<{E}_{0, surf}^{t, def}<{E}_{0, surf}^m$ [18].

In the region of vibrations of the surface groups Zr = O (OH) there is a broad band. The main contributions to the intensities of this band are absorption bands with peaks at 1015 cm^-1^, 1040 cm^-1^, 1088 cm^-1^, and 1171 cm^-1^. It should be noted that the main contribution to this submaximum of the IR spectrum is made by the absorption band at 1088 cm^-1^ for all presented systems. At the same time for physically and/or chemically modified systems, there is an increasing in the contribution to the submaximum of the low-frequency bands. These changes may be related to the reorganization of the nanoparticles surface that occurred as a result of the martensitic tetragonal-monoclinic transformation, and as a result of a changing in the surface defect under the action of modifying factors.

In the frequency range 1700–1200 cm^-1^, the bands of deformation vibrations of OH bonds of water molecules coordinated on the surface of oxide nanoparticles (1638 and 1557 cm^-1^) are observed for the initial system. The appearance in the IR spectrum of modified systems of the absorption band at 1737 cm^-1^ and a number of bands in the low-frequency part of this range indicates the process of surface carbonization under the conditions of modification, especially of a physical nature. A number of absorption bands of 2960, 2925, and 2856 cm^-1^ correspond to the C-H bonds of the aliphatic groups CH_3_ and CH_2_ indicating the presence of a small amount of organic matter on the surface of the nanoparticles.

A wide absorption band of 3700–3200 cm^-1^ is due to stretching vibrations of the OH bond of water molecules coordinated on the surface of the particles. It should be note that in the case of systems both original ZrO_2_-Y_2_O_3_ and chemically modified ZrO_2_-Y_2_O_3_-SiO_2_ processed by mechanical action the intensification of the low-frequency shoulder of this band is observed which indicates the formation of identical active centers on the particles surface of physically modified systems.

Thus, the detected features of the IR spectrum of physically and/or chemically modified systems indicate a changing in the surface state of the particles that leads to a change in their surface energy and as a consequence to the reactivity of the particles.

**Fig. 3:**
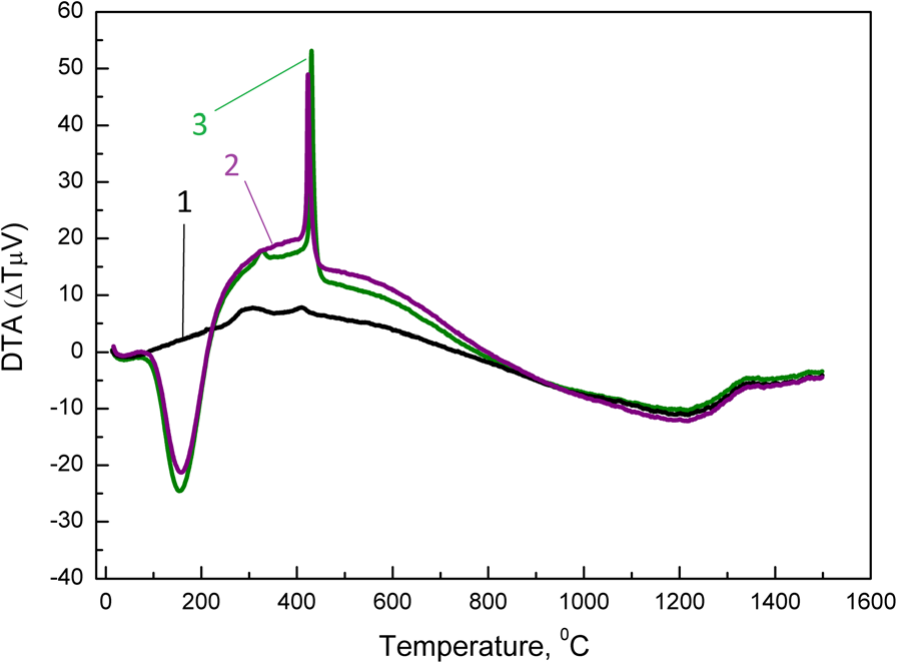
DTA curves of samples: 1- PMM8-3Y-TZP-0.2 wt% SiO_2_(nanopowders were calcined at 1000 °C for 2 h); 2 - 3Y-TZP-0.2 wt% SiO2 (hydroxide); 3- 3Y-TZP (hydroxide)

The thermal characteristics of nanopowders were analyzed using DTA instrument. The nanopowders were heated to the 1500 °C with heating rate of 10 °C/min. Figure 3 shows DTA curves of the nanopowders obtained by co-precipitation and mixing methods with and without silica. The endothermic peak in DTA curve (around 157 °C) was determined to the evaporation of physical water in the amorphous gel. The exothermic peaks (around 423 and 430 °C) in curves of both nanopowders with and without silica were assigned to the crystallization. As seen from Fig. 3, the silica additive has almost no effected on the dynamics of crystallization processes.

**Fig. 4:**
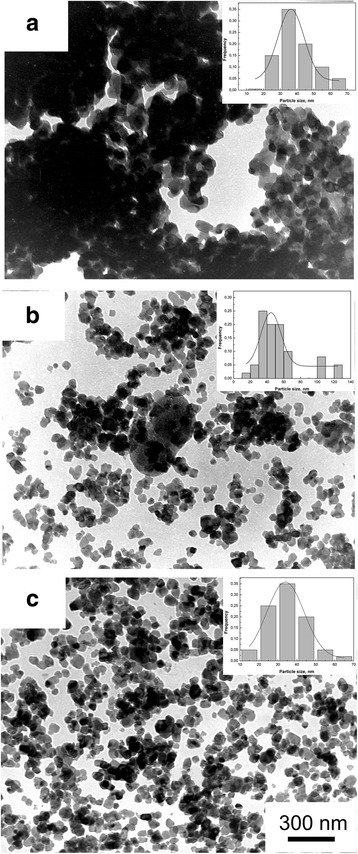
TEM images and histograms of particle size distribution of (**a**) 3Y-TZP, **b** PM4-3Y-TZP, **c** PM8-3Y-TZP nanopowders

**Fig. 5:**
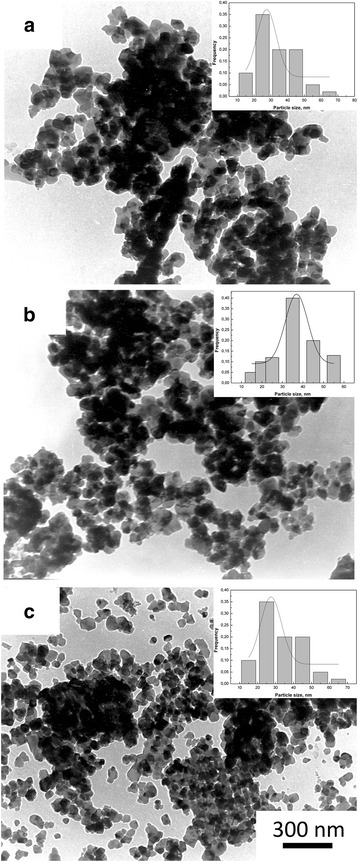
TEM images and histograms of particle size distribution of (**a**) 3Y-TZP-0.2 wt% SiO_2_, **b** PMM4-3Y-TZP-0.2 wt% SiO_2_, **c** PMM8-3Y-TZP-0.2 wt% SiO_2_ nanopowders

**Fig. 6:**
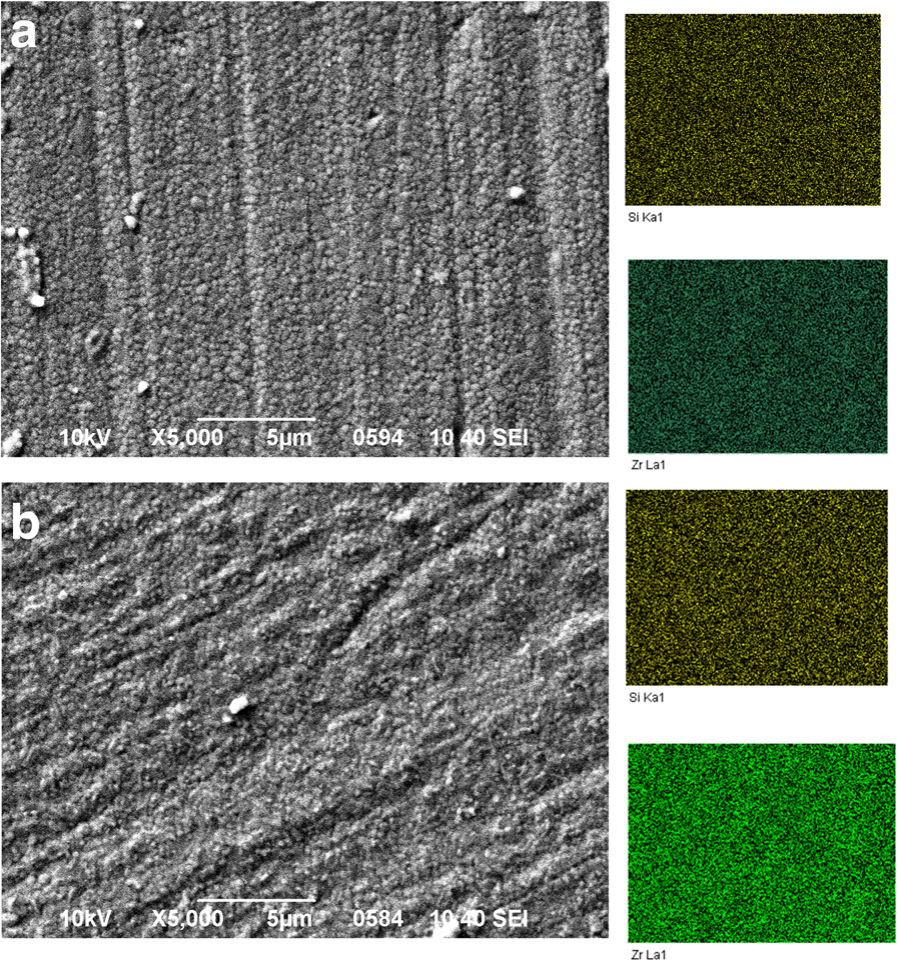
The SEM images and EDX mapping analysis of sintered to 1500 °C samples (**a**) 3Y-TZP-0.2 wt% SiO_2_, and **b** PMM8-3Y-TZP-0.2 wt% SiO_2_

In Fig. 4 are shown TEM images of 3Y -TZP nanopowders with (b, c) and without milling (a). As can be seen 3Y-TZP (a) has a sufficiently high aggregation degree. However, it should be noted the aggregates are “soft” and can be easily destroyed by a mechanical action, observed after 4 h and after 8 h of milling. Figure 5 has shown TEM images of the structure and the histograms of particle size distribution of 3Y-TZP nanopowders with silica, which was obtained by co-precipitation (a) and by mixing method with milling for 4 h (b) and 8 h (c). The effect of silica additive and mechanical activation on the particle sizes and agglomeration degree (agglomeration factor F_a_, %) of nanopowders are shown in Table 2. The silica additive insignificantly affected the specific surface area. Mechanical milling leaded to the increase S_BET_ with increasing the milling time. The maximum surface area was achieved in the powder PMM8-3Y-TZP + 0.2 wt% SiO_2_. The agglomeration degree decreases at the 4-h milling. But in this case, the silica additive distributed in the 3Y-TZP unevenly. The important result is that 8 h of milling were enough for the additive to distribute on the surface of the 3Y-TZP in the best possible way. As seen from the Fig. 6, such small silica additive distributed in ceramic structure uniform independently of dopant addition method.

**Fig. 7:**
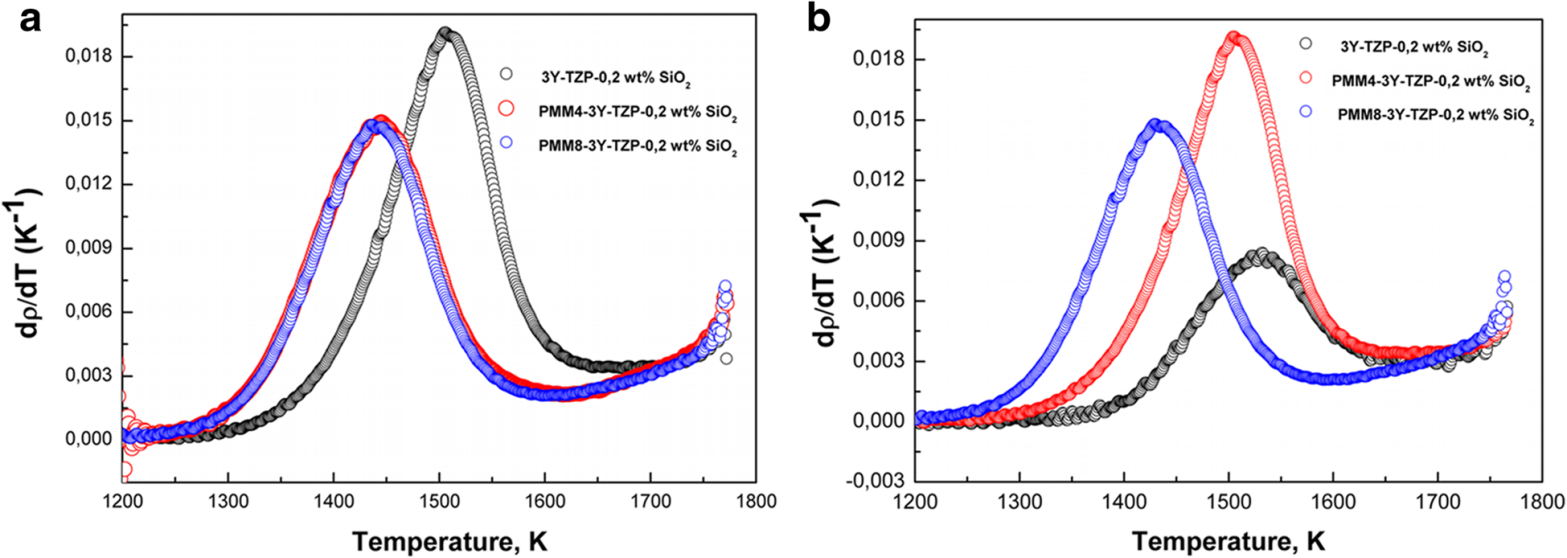
Temperature dependence of densification rates of (**a**) 3Y-TZP, PM4-3Y-TZP, PM8-3Y-TZP and **b** 3Y-TZP-0.2 wt% SiO_2_, PMM4-3Y-TZP-0.2 wt% SiO_2_, PMM8-3Y-TZP-0.2 wt% SiO_2_ nanopowders

The temperature dependence of densification rates (dρ/dT) of the 3Y-TZP nanopowders with and without milling is shown in the Fig. 7. As we can see the nanopowder 3Y-TZP without milling achieved the maximum densification rate at a lower temperature than the milling nanopowders. For these powders, the densification curves shifted to the higher temperature.

**Fig. 8:**
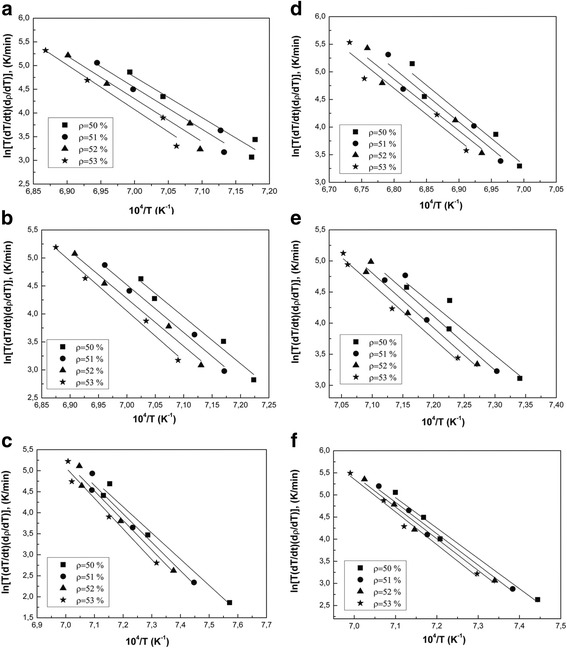
Arrhenius-type plots of (**a**) 3Y-TZP, **b** PM4-3Y-TZP, **c** PM8-3Y-TZP, **d** 3Y-TZP-0.2 wt% SiO_2_, **e** PMM4-3Y-TZP-0.2 wt% SiO_2_, and **f** PMM8-3Y-TZP-0.2 wt% SiO_2_ nanopowders

As Arrhenius-type plot (Fig. 8) and Table 3 shows the powder 3Y-TZP without milling is already sintered by volume diffusion mechanism. That is why it is sintered faster than others. It is a goal that other researchers [3–7] wish to achieve on their nanopowders using various additives, including silica. However, we have already achieved this goal due to our unique nanopowders production technology.

**Table 3:** Sintering mechanisms, parameters *n* and activation energies *Q* of investigated nanopowders

No.	Nanopowders	*n*	Q (kJ/mol)	Sintering mechanism
1	3Y-TZP	1/2	667	VD
2	4PM- 3Y-TZP	1/2	747	VD
3	8PM- 3Y-TZP	1/3	804	GBD
4	3Y-TZP-0.2 wt % SiO_2_	1/3	830	GBD
5	4PMM-3Y-TZP-0.2 wt % SiO_2_	1/2	680	VD
6	8PMM-3Y-TZP-0.2 wt % SiO_2_	1/2	589	VD

The biggest mechanical activation effect was achieved at 8 h of milling. In this case, the sintering mechanism changed from VD to GBD. It is a well-known fact that the initial Y-TZP (Tosoh) powders are produced by hydrolysis with the milling time of 48 h (patent JP 3680338). As a result of our previous investigation, the milling time can be reduced from 48 h to only 4 and 8 h. Under the same conditions but using our (DIPE) nanopowders we have managed to save time [8, 9]. It was found that 8 h of milling is more than enough for the powders under analysis to change the sintering mechanism from VD to GBD.

As for the powders with silica additives the maximum densification rate of the nanopowders PMM4-3Y-TZP-0.2 wt% SiO_2_, PMM8-3Y-TZP-0.2 wt% SiO_2_ obtained using the mixing method was achieved at lower temperature than in the sample 3Y-TZP-0.2 wt% SiO_2_ prepared by co-precipitation. This means that they are sintered faster. And in this case, a dominant mechanism at the initial sintering stage was VD mechanism. In contrast, the powders 3Y-TZP with 0.2 wt% SiO2 obtained by co-precipitation were sintered due to the predominance of the GBD mechanism.

## Conclusions

It was shown the impact of the silica additive, various ways of silica addition and mechanical activation on the kinetics of the initial sintering stage. The following conclusions were obtained:

1. The silica additive is the reason for the change in the predominant mechanism at the initial sintering stage from VD to the GBD in nanopowders obtained by co-precipitation. It should be noted that such a small amount (only 0.2 wt% SiO_2_) has a strong influence on the sintering kinetics.
2. In case of nanopowders obtained by mixing, since the processes occur with the predominance of the VD mechanism, the sum total of both factors—the silica additive and the mechanical activation—leads to the acceleration of the sintering.
3. The mechanical activation also causes a change in the sintering mechanism from VD to GBD; and here, the most important issue is the milling time (just an 8-h milling).

## Abbreviations

3Y-TZP: 3 mol % yttria-stabilized tetragonal zirconia polycrystal
CRH: Constant rate of heating
DIPE: Donetsk Institute for Physic and Engendering
GBD: Grain-boundary diffusion
NAS: National Academy of the Science
PM4 and PM8: Powder milling during for 4 and 8 hours
PMM4 and PMM8: Powder mixing and milling during for 4 and 8 hours
S_BET_: Specific surface area measured by the Brunauer-Emmett-Teller (BET) method
TEM: Transmission electron microscopy
VD: Volume diffusion
Wt %: Weight percent
XRD: X-ray diffraction
